# Case Report: Pharmacogenetics Applied to Precision Psychiatry Could Explain the Outcome of a Patient With a New *CYP2D6* Genotype

**DOI:** 10.3389/fpsyt.2021.830608

**Published:** 2022-02-25

**Authors:** Elena Marcos-Vadillo, Lorena Carrascal-Laso, Ignacio Ramos-Gallego, Andrea Gaedigk, Belén García-Berrocal, Eduardo Mayor-Toranzo, Alfonso Sevillano-Jiménez, Almudena Sánchez, María Isidoro-García, Manuel Franco-Martín

**Affiliations:** ^1^Servicio de Bioquímica, Hospital Universitario de Salamanca, Instituto de Investigacion Biomedica de Salamanca, Salamanca, Spain; ^2^Servicio de Psiquiatría, Hospital Provincial de Zamora, Instituto de Investigacion Biomedica de Salamanca, Zamora, Spain; ^3^Departamento de Fisiología y Farmacología, Universidad de Salamanca, Salamanca, Spain; ^4^Division of Clinical Pharmacology, Toxicology and Therapeutic Innovation, Children's Mercy Kansas City, Kansas City, MO, United States; ^5^Department of Pediatrics, School of Medicine, University of Missouri-Kansas City, Kansas, MO, United States; ^6^Servicio de Farmacia, Hospital Universitario de Salamanca, Instituto de Investigacion Biomedica de Salamanca, Salamanca, Spain; ^7^Departamento de Medicina, Universidad de Salamanca, Salamanca, Spain

**Keywords:** antipsychotic agents, pharmacogenetics, cytochrome P450 enzyme system, psychotic disorders, precision medicine

## Abstract

Precision medicine applied to psychiatry provides new insight into the promising field of precision psychiatry. Psychotic disorders are heterogeneous, complex, chronic, and severe mental disorders. Not only does the prognosis and the course of the disease vary among patients suffering from psychotic disorders, but the treatment response varies as well. Although antipsychotic drugs are the cornerstone of the treatment of schizophrenia, many patients only partially respond to these drugs. Furthermore, patients often experience adverse events which can lead to poor treatment adherence. Interindividual variability in drug response could be related to age, gender, ethnicity, lifestyle factors, pharmacological interactions, obesity, and genetics, all of which influence the process of drug metabolism. Commonly prescribed antipsychotics are metabolized by cytochrome P450 (*CYP450*) enzymes, and *CYP450* genes are highly polymorphic. Pharmacogenetic testing is increasingly being used to predict a patient's drug response and could help to find the most appropriate therapy for an individual patient. In this report, we describe a psychotic patient who did not receive adequate clinical follow-up and subsequently presented adverse events, which could be explained by his pharmacogenetic profile and the drug interactions resulting from the polypharmacy prescribed.

## Introduction

An increasing number of children, adolescents, and adults (1) are being diagnosed with mental illness, including depressive disorder, bipolar disorder, and schizophrenia spectrum disorder (2–7). According to the World Health Organization (8), people with mental disorders experience higher rates of disability and mortality compared to the general population, making these conditions a priority of health systems.

Psychotic disorders are a group of heterogeneous, complex, chronic, and severe mental diseases that manifest through positive (hallucinations, delusions, etc.) and negative (impaired motivation, social withdrawal, etc.) symptoms, affecting approximately 1% of the global population (9, 10). Not only are the prognosis and disease course of psychotic disorders variable among patients, but so is treatment response. Antipsychotic drugs comprise the fundamental treatment of schizophrenia and other psychotic disorders (11, 12), but many patients do not only partially respond to drug therapy (13, 14). Furthermore, patients often experience side effects when taking antipsychotics including sedation, cognitive impairment, weight gain and obesity, metabolic syndrome, cardiovascular and neuromotor side effects, among others, which can lead to poor adherence or discontinuation of treatment (15–19).

Interindividual variability in the hepatic metabolism of these drugs may be impacted by several factors including age, gender, ethnicity, lifestyle factors (e.g., tobacco and alcohol consumption, diet), pharmacological interactions (patients taking multiple drugs), obesity, and variation in the genes contributing to the metabolism of the drugs taken (20–22). Many commonly prescribed antipsychotics are metabolized by cytochrome P450 enzymes, mainly *CYP2C19* and *CYP2D6* (23–26).

*CYP* genes are highly polymorphic, and patients with extreme phenotypes, i.e., those with no or low activity or increased activity, have been shown to have an increased risk of adverse events or treatment failure due to drug levels that are either too high or too low (27, 28). Thus, pharmacogenetic testing is increasingly utilized to predict a patient's metabolic capacity and utilize this knowledge to inform drug therapy (29–33). Variable enzyme activity can to a large part be explained by the presence of variations on the nucleotide level [i.e., single-nucleotide polymorphisms (SNPs)] but also be caused by gene copy number variation (CNVs), i.e., gene duplications or deletions, and rearrangements (34, 35). Genotype is typically translated into phenotype [i.e., poor metabolizers (PM), intermediate metabolizers (IM), normal metabolizers (NMs), rapid metabolizers (RMs), and ultrarapid metabolizers (UMs)] and therapeutic recommendations based thereof. For *CYP2D6* genotype to phenotype translation, recommendations have been published by the Clinical Pharmacogenetics Implementation Consortium (36). Additional details and translation tables for other *CYPs* can be found on the PharmGKB website at https://www.pharmgkb.org/page/pgxGeneRef.

*CYP2D6* is one of the most important and extensively studied *CYP450* enzymes (34, 37). It has been shown to contribute to the metabolism of over 20% of commonly prescribed medications (38) including atypical antipsychotics such as aripiprazole, asenapine, clozapine, olanzapine, quetiapine, and risperidone. To date, the Pharmacogene Variation Consortium (39, 40) has described over 140 variant alleles (star alleles) (41). Approximately 5–10% of the European population are poor metabolizers due to having two nonfunctional alleles (42). Additional information regarding allele and genotype frequencies can be found on the PharmGKB website.

In this report, we describe a psychotic patient who did not receive adequate clinical follow-up and presented with side effects, which could have been prevented considering pharmacogenetic test results, complex history, and polypharmacy.

## Case Description

**Figure 1:**
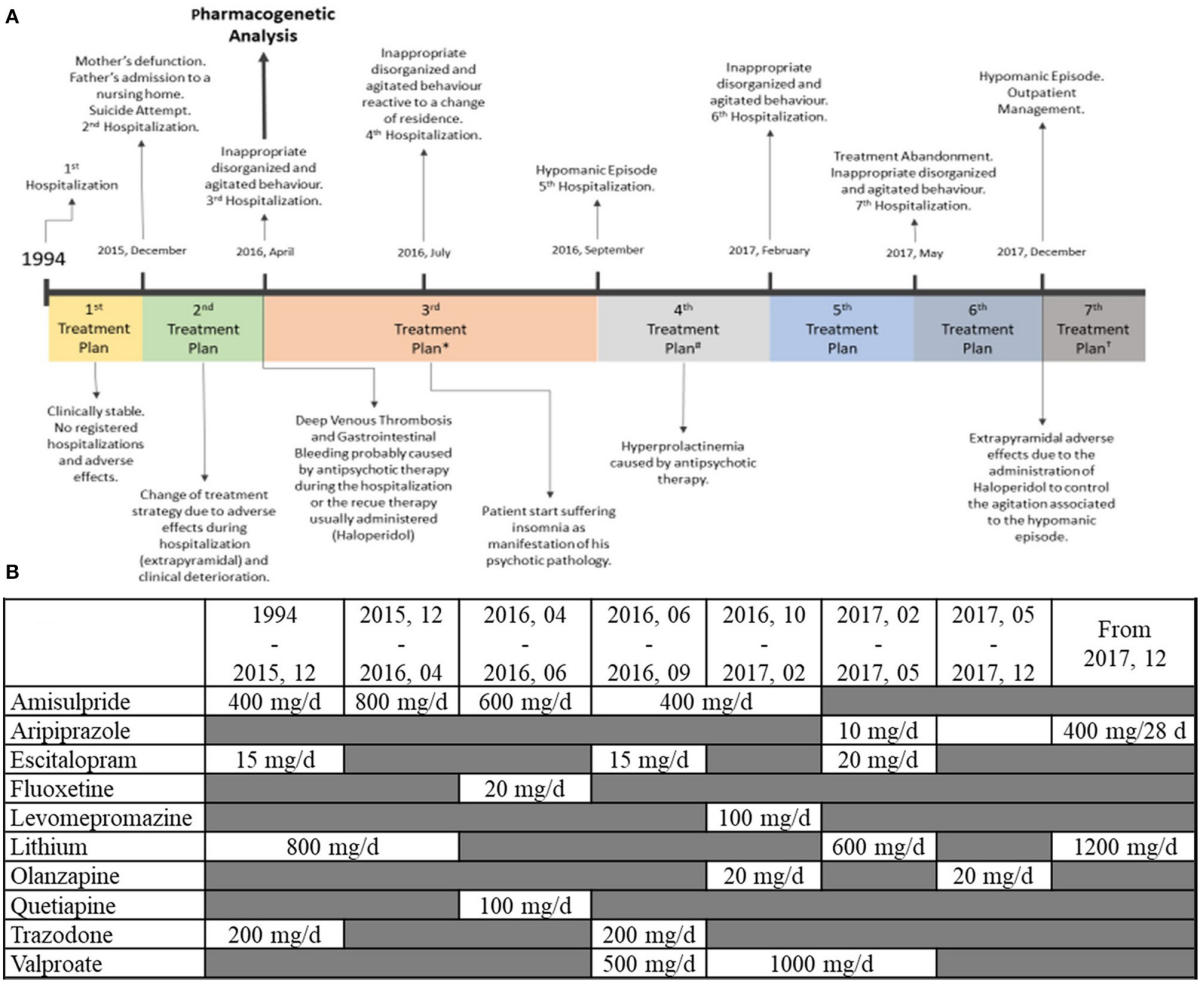
**(A)** Clinical history of the patient from 1994 to the end of the case study. *Therapeutic plan is slightly modified due to the patient's complaints of insomnia. #One of the antipsychotics used is discontinued due to the appearance of hyperprolactinemia.^†^After stabilizing a hypomanic episode, olanzapine (oral) is substituted by aripiprazole (LAI), and therapy is readjusted to control insomnia. **(B)** Pharmacotherapy doses. Daily doses administered through the different stages of the patient clinical evolution. Mg, milligram; D, day.

The following report describes the situation of a male European patient, who gave us consent to publish his situation, who was born in 1956, and who had been receiving mental health care since 1993, initially under private care, with a diagnosis of schizotypal personality disorder (DSM-V, F21). Regarding the psychiatric family history, his father suffered from bipolar disorder (DSM-V, F31), and his brothers suffered from major depressive disorder (DSM-V, F33). The review of the family history also revealed a psychotic disorder in a second-degree relative (niece). Somatic personal history included hemorrhoids and an anal fissure. The patient did not smoke, drink alcohol, or have a history of substance abuse. During the routine clinical analysis performed throughout the study, there were no ionic, metabolic, or hematological alterations found. In 1994, during his first admission at the University Hospital of Salamanca, he was diagnosed with schizoaffective disorder bipolar type (DSM-V, F25.0). From 1994 to 2015, follow-up treatment was carried out with no hospital admissions and no documented adverse events (AE). In the months following his mother's death and the admission of this father into a care facility, the patient overdosed on lithium with autolytic intention, which required a 3-day stay in intensive care. After this event, the patient clinically deteriorated, and his treatment was changed in seven phases (see Figure 1A):

**1st treatment plan (1994–18/01/2016)**: The first admission was triggered by hypomanic symptoms (expansive mood, ideo-fleeting speech, decreased sleep needs, and maniform symptomatology), after which he was diagnosed with schizoaffective disorder. The treatment prescribed when he was described was as follows: lithium, amisulpride, escitalopram, trazodone, and clobazam. No documented AE. After a sudden change in his familiar environment, the patient overdosed on lithium, with autolytic intention, which required a 3-day stay in intensive care, and was hospitalized for 43 days (6/12/2015–18/01/2016), presenting with rudeness, inappropriate sexual behavior, childish utterances, emotional coldness, and antagonistic behavior. Previous treatment was continued with minor extrapyramidal effects. For pharmacogenetic interactions (see Figure 1B).
**2nd treatment plan (18/01/2016–13/06/2016):** Based on the adverse effect referred by the patient and taking into account his poor clinical progress during the last hospitalization, the prescriber adjusted the drug treatment upon the patient's release to only include lithium and amisulpride, neither of which is metabolized by a *CYP* enzyme. Although this adjustment reduced adverse effects, the patient's clinical condition did not improve, and he was readmitted for 49 days (25/04/2016–13/06/2016) due to disturbed behavior with neither depressive nor psychotic symptomatology whereupon he was treated with amisulpride, lorazepam, lormetazepam, quetiapine, and fluoxetine with deep venous thrombosis and gastrointestinal bleeding (melena) as AE.
  A pharmacogenetic test [AmpliChip *CYP450* Test (43), the Antigenomics platform, MassARRAY 4.2 (Agena), and probed-based assays using the Light-Cycler platform] was performed while the patient was hospitalized. The following allelic variants were tested: *CYP2B6* (^*^6), *CYP2C9* (^*^2 and ^*^3), *CYP2C19* (^*^2, ^*^3, and ^*^17), *CYP2D6* (^*^2, ^*^3, ^*^4, ^*^5, ^*^6, ^*^7, ^*^8, ^*^9, ^*^10, ^*^12, ^*^14, ^*^17, ^*^29, ^*^41, and presence of gene duplication), *CYP3A4* (−*392 G>A*), *CYP3A5* (^*^3), and *MDR1* (3435C>T) (Table 1). Allele nomenclature was per the Pharmacogenetic Variation (PharmVar) Consortium (41). The *CYP2D6* genotype, however, was returned as a “no call”. Subsequent gene resequencing revealed that the patient had a novel allele, which was submitted to PharmVar (41) and designated CYP2D6^*^119. The genotype of the patient was revised to CYP2D6^*^4/^*^119. The CYP2D6^*^119 allele shares many sequence variants with the decreased function CYP2D6^*^41 allele but lacks 2851C>T (p.R296C). Due to the presence of 2989G>A which has been shown to impact splicing, the CYP2D6^*^119 allele may have decreased function (PMID 33043448); thus we classified the patient as an IM (it is noted though that CPIC classified this alleles as “unknown function”). PM status for *CYP3A5* and decreased expression of the *MDR1* drug transporter were also discovered.
**3rd treatment plan (13/06/2016–04/10/2016):** Upon discharge, a treatment readjustment was made, as the potential increase in quetiapine plasma levels due to the quetiapine-fluoxetine pharmacokinetic interaction was thought to be related to the emergence of the deep venous thrombosis, combined with the fact that the hospital stay was prolonged, and the patient could have been bedridden longer than usual. The new prescription consisted of lorazepam, amisulpride, valproate, escitalopram, and trazodone. There were no documented AE, but required two hospitalizations of 9 days (04/07/2016–13/07/2016), due to behavioral disturbances in response to a change of residence, and 21 days (13/09/2016–04/10/2016), due to an exacerbation of the hypomanic symptomatology. Previous treatment was continued with minor extrapyramidal effects and insomnia.
**4th treatment plan (October 2016–February 2017):** Given the profile of adverse effects reported by the patient (excessive morning sedation and extrapyramidal symptoms) and the fact that the patient was not receiving a satisfactory therapeutic benefit, the pharmacological treatment was readjusted to amisulpride, olanzapine, valproate, and levomepromazine. Hyperprolactinemia was found in a routine clinical analysis, and amisulpride was suspended. The patient was hospitalized for 9 days (22/02/2017–03/03/2017) because of behavioral disturbances and depressed mood with delusions of guilt and harm, exhibiting mutism and a catatonic attitude. Treatment was continued with extrapyramidal effects during the hospitalization.
**5th treatment plant (February 2017–May 2017):** The patient was discharged with aripiprazole, lithium, clonazepam, lormetazepam, and citalopram. Minor extrapyramidal effects. Hospitalized for 12 days (06/05/2017–18/05/2017), due to disorganized behavior and refusal to take medication, with olanzapine, citalopram, lithium, clonazepam, and lormetazepam.
**6th treatment plant (May 2017–December 2017):** At discharge, the patient continued with olanzapine, clonazepam, lormetazepam, and lorazepam. The patient presented extrapyramidal symptoms after administration of haloperidol due to a manic episode managed as an outpatient.
**7th treatment plant (from December 2017):** Given the need to control the patient's sleep pattern and in light of the sedative effects of olanzapine, olanzapine was replaced with aripiprazole, eventually a long-acting injectable (LAI), to ensure adherence and simplify the absorption variables, complementing the therapy with lithium, clonazepam, lormetazepam, and pregabalin. From this point, the patient did not present AE, nor needed hospitalization.

**Table 1:** Pharmacogenetic analysis.

**Gene**	**Alleles tested**	**Genotype**	**Predicted phenotype**
*CYP2C19*	^*^2, ^*^3, ^*^17	^*^1/^*^1	Normal metabolizer
*CYP2D6*	2-^*^10, ^*^12, ^*^14, ^*^17, ^*^29, ^*^41, XN	^*^4/^*^119	Intermediate metabolizer
*CYP2C9*	^*^2, ^*^3	^*^1/^*^1	Normal metabolizer
*CYP2B6*	^*^6	^*^1/^*^1	Normal metabolizer
*CYP3A4*	−392 G>A	A/A	Normal metabolizer
*CYP3A5*	^*^3	^*^3/^*^3	Poor metabolizer
*MDR1*	3435C>T	C/T	Decreased expression

*Alleles listed using the PharmVar Haplotype nomenclature*.

* *Allele*.

*Phenotype predictions are according to those recommend by CPIC which are summarized by respective “diplotype to phenotype translation” tables (available at: https://www.pharmgkb.org/page/pgxGeneRef)*.

## Discussion

We present a patient with a novel *CYP2D6* allele, CYP2D6^*^119, which was paired with a known non-functional allele. Since the novel allele and CYP2D6^*^41, a known decreased function allele, essentially only differ by an SNP that is believed to not alter the enzymatic activity, we classified the patient as IM. This phenotype (i.e., decreased *CYP2D6* activity) may have contributed to the extrapyramidal AE presented after administration of levomepromazine and haloperidol, and the sedation associated with olanzapine. *CYP2D6* has also been described to contribute to the metabolism of quetiapine and aripiprazole. Furthermore, the patient's *ABCB1* genotype may have contributed to the hyperprolactinemia associated with amisulpride, to the deep venous thrombosis related to quetiapine, and, together with *CYP2D6*, to the sedation due to olanzapine. While *CYP2D6* and *CYP3A5* are not always the major metabolic pathway of drugs associated with AE presented by the patient, there could be a phenocopy effect related to drugs metabolized by these pathways.

This complex case illustrates how AE can impact patient compliance (44), as shown in two hospitalizations (February 2017, May 2017) caused by the patient refusing to continue the treatment. The patient was prescribed antipsychotics, which appeared to have repeatedly caused AE. Pharmacogenetic testing helped to partially understand the origin of his AE; it was possibly caused by a combination of his pharmacokinetic phenotype (reduced *CYP2D6* and *CYP3A5* enzymatic activity and *MDR1* expression) and the phenocopy effect related to pharmacokinetic interactions (Table 2). A treatment plan that was guided by the patient's pharmacogenetic profile and knowledge about the pharmacokinetics of drugs prescribed drastically improved his medical condition. The patient ultimately reported no AE and continued to evolve favorably underscoring the utility of pharmacogenetic testing to guide pharmacotherapy. The application of this methodology could ensure a significant reduction in both pharmaceutical (fewer exacerbations hence less rescue medication, lower dosages, etc.) and hospitalization-related (fewer hospitalizations required to manage adverse effects or achieving better treatment adherence due to the reduction in the emergence of these adverse effects) economical costs. Although the evolution of the patient is not certain, the clinical intervention applied has a lifelong nature, and the results obtained by the pharmacogenetic analysis could be used by other medical areas. These conclusions should be considered taking into account the limitations of this study. Being this case report based on the application of a recently implemented approach to our service, we could not make use of all the possible variables that could be helpful when managing these patients (i.e., antipsychotic plasma levels). Moreover, this methodology was introduced, concurring with the increasing use of LAI presentations, which facilitate therapeutic adherence, and it could be a possible confounder. One of the strong points of this approach is that the decisions concerning the pharmacotherapy of the patient were agreed to by a multidisciplinary team (in which psychiatrists and clinical biochemists, among other clinical specialties, participated), which permitted a more profound knowledge about the drug metabolism and the factors influencing drug varying factors and promoted education on this subject for the bio-sanitary personnel (including psychiatric, nurses, psychologist, etc.).

**Table 2:** Pharmacogenetic interaction of all treatment of the patient.

	**Cytochrome P450**										
	**1A2**	**2B6**	**2C8**	**2C9**	**2C19**	2D6 (^*^)	**2E1**	**3A4**	**3A5 (^†^)**	**3A7**	**MDR1 (^‡^)**
Amisulpride											S
Aripiprazole						S (+), Ih Id		S (+)	S (-)	S (-)	S
Escitalopram					S (+)	S (-) Ih		S (-)			S
Fluoxetine	S, Ih	S, Ih	S, Ih	S (+) Ih	S (+) Ih	S (+) Ih	S	S (+) Ih			S
Levomepromazine						Ih, S (+)	Ih				
Lithium											
Olanzapine	S (+)			Ih	Ih	S (±) Ih		Ih			S
Quetiapine					S (-)	S (±) Ih		S (+)	S (-)	S (-)	
Trazodone						S (+)		S (+)	S (-)	S (-)	
Valproate			Ih	S (+) Ih	S (-) Ih			Ih			Id

* CYP2D6*4/*119, CYP2D6 predicted intermediate metabolizer (IM) phenotype.

† CYP3A5*3/*3 predicted poor metabolizer (PM) phenotype.

‡ *MDR1 3435C/3435T predicted decreased expression*.

*Id (Inducer). Ih (Inhibitor). S (Substrate). + (Major Metabolic Pathway), ± (Minor Metabolic Pathway), - (Minor Metabolic Pathway likely not clinically relevant)*.

## Data Availability Statement

The datasets presented in this study can be found in online repositories. The names of the repository/repositories and accession number(s) can be found in the article/supplementary material.

## Ethics Statement

The studies involving human participants were reviewed and approved by Ethics Committee of the University Hospital of Salamanca (CEIC ref.: 107/ 12/2016). The patients/participants provided their written informed consent to participate in this study.

## Author Contributions

AG, MI-G, and MF-M: conceptualization. EM-V, LC-L, and MI-G: methodology. LC-L and IR-G: software. MI-G, BG-B, and EM-T: validation. EM-V, LC-L, and IR-G: formal analysis, investigation, data curation, and writing-original draft. EM-T and AS-J: resources. AG, AS, MI-G, and MF-M: writing-review and editing. LC-L, IR-G, and MI-G: visualization. MI-G and MF-M: project administration. All authors contributed to the article and approved the submitted version.

## Conflict of Interest

The authors declare that the research was conducted in the absence of any commercial or financial relationships that could be construed as a potential conflict of interest.

## Publisher's Note

All claims expressed in this article are solely those of the authors and do not necessarily represent those of their affiliated organizations, or those of the publisher, the editors and the reviewers. Any product that may be evaluated in this article, or claim that may be made by its manufacturer, is not guaranteed or endorsed by the publisher.

## References

[1] Hálfdánarson Ó Zoëga H Aagaard L Bernardo M Brandt L Fusté AC. International trends in antipsychotic use: a study in 16 countries, 2005-2014. Eur Neuropsychopharmacol. (2017) 27:1064–76. 10.1016/j.euroneuro.2017.07.001 28755801

[2] McGrath J Saha S Chant D Welham J. Schizophrenia: a concise overview of incidence, prevalence, and mortality. Epidemiol Rev. (2008) 30:67–76. 10.1093/epirev/mxn001 18480098

[3] Rintala H Chudal R Leppämäki S Leivonen S Hinkka-Yli-Salomäki S Sourander A. Register-based study of the incidence, comorbidities and demographics of obsessive-compulsive disorder in specialist healthcare. BMC Psychiatry. (2017) 17:64. 10.1186/s12888-017-1224-3 28183286 PMC5301466

[4] Rao P Moore JK Stewart R Runions K Bear N Wong JWY. Bipolar disorder in children and adolescents: diagnostic inpatient rates from 2000 to 2013 in Germany. Int J Bipolar Disord. (2016) 4:23. 10.1186/s40345-016-0064-2 27837521 PMC5106426

[5] Okkels N Vernal DL Jensen SOW Mcgrath JJ Nielsen RE. Changes in the diagnosed incidence of early onset schizophrenia over four decades. Acta Psychiatr Scand. (2012) 127:62–8. 10.1111/j.1600-0447.2012.01913.x 22906158

[6] Kühl JOG Laursen TM Thorup A Nordentoft M. The incidence of schizophrenia and schizophrenia spectrum disorders in Denmark in the period 2000-2012. A register-based study. Schizophr Res. (2016) 176:533–9. 10.1016/j.schres.2016.06.023 27341953

[7] Olfson M Druss BG Marcus SC. Trends in mental health care among children and adolescents. N Engl J Med. (2015) 372:2029–38. 10.1056/NEJMsa1413512 25992747

[8] WHO. Mental Health Action Plan 2013-2020 WHO. World Health Organization (2015). Available online at: http://www.who.int/entity/mental_health/publications/action_plan/en/index.html (accessed November 25, 2021).

[9] Henriksen MG Nordgaard J Jansson LB. Genetics of schizophrenia: overview of methods, findings and limitations. Front Hum Neurosci. (2017) 11:322. 10.3389/fnhum.2017.00322 28690503 PMC5480258

[10] Owen MJ Sawa A Mortensen PB. Schizophrenia. Lancet. (2016) 388:86–97. 10.1016/S0140-6736(15)01121-6 26777917 PMC4940219

[11] Lally J MacCabe JH. Antipsychotic medication in schizophrenia: a review. Br Med Bull. (2015) 114:169–79. 10.1093/bmb/ldv017 25957394

[12] Ozomaro U Wahlestedt C Nemeroff CB. Personalized medicine in psychiatry: problems and promises. BMC Med. (2013) 11:132. 10.1186/1741-7015-11-132 23680237 PMC3668172

[13] Martin A Downing J Maden M Fleeman N Alfirevic A Haycox A. An assessment of the impact of pharmacogenomics on health disparities: a systematic literature review. Pharmacogenomics. (2017) 18:1541–50. 10.2217/pgs-2017-0076 29095091 PMC5694021

[14] Ravyn D Ravyn V Lowney R Nasrallah HA. *CYP450* Pharmacogenetic treatment strategies for antipsychotics: a review of the evidence. Schizophr Res. (2013) 149:1–14. 10.1016/j.schres.2013.06.035 23870808

[15] Solmi M Murru A Pacchiarotti I Undurraga J Veronese N Fornaro M. Safety, tolerability, and risks associated with first- and second-generation antipsychotics: a state-of-the-art clinical review. Ther Clin Risk Manag. (2017) 13:757–77. 10.2147/TCRM.S117321 28721057 PMC5499790

[16] Lieberman JA Stroup TS McEvoy JP Swartz MS Rosenheck RA Perkins DO. Effectiveness of antipsychotic drugs in patients with chronic schizophrenia. N Engl J Med. (2005) 353:1209–23. 10.1056/NEJMoa051688 16172203

[17] Foster A. Pharmacogenetics of antipsychotic adverse effects: case studies and a literature review for clinicians. Neuropsychiatr Dis Treat. (2008) 3:965–73. 10.2147/NDT.S1752 19300635 PMC2656342

[18] Shenoy S R A Bhandary RP Praharaj SK. Frequency, reasons, and factors associated with antipsychotic polypharmacy in Schizophrenia: a retrospective chart review in a tertiary hospital in India. Asian J Psychiatr. (2020) 51:102022. 10.1016/j.ajp.2020.102022 32278888

[19] Li N Cao T Wu X Tang M Xiang D Cai H. Progress in genetic polymorphisms related to lipid disturbances induced by atypical antipsychotic drugs. Front Pharmacol. (2020) 10:1669. 10.3389/fphar.2019.01669 32116676 PMC7011106

[20] Eum S Lee AM Bishop JR. Pharmacogenetic tests for antipsychotic medications: clinical implications and considerations. Dialogues Clin Neurosci. (2016) 18:323–37. 10.31887/DCNS.2016.18.3/jbishop 27757066 PMC5067149

[21] Zhuo C Hou W Lin C Hu L Li J. Potential value of genomic copy number variations in schizophrenia. Front Mol Neurosci. (2017) 10:204. 10.3389/fnmol.2017.00204 28680393 PMC5478687

[22] Mahintamani T Mitra S Kavoor A Nizamie Sh. Negative symptoms in schizophrenia. Ind Psychiatry J. (2016) 25:135. 10.4103/ipj.ipj_30_15 28659691 PMC5479085

[23] Urichuk L Prior T Dursun S Baker G. Metabolism of atypical antipsychotics: involvement of cytochrome P450 enzymes and relevance for drug-drug interactions. Curr Drug Metab. (2008) 9:410–8. 10.2174/138920008784746373 18537577

[24] Lynch T Price A. The effect of cytochrome P450 metabolism on drug response, interactions, and adverse effects. Am Fam Physician. (2007) 76:391–6. 17708140

[25] Sheehan JJ Sliwa JK Amatniek JC Grinspan A Canuso CM. Atypical antipsychotic metabolism and excretion. Curr Drug Metab. (2010) 11:516–25. 10.2174/138920010791636202 20540690

[26] Hoffmann MF Preissner SC Nickel J Dunkel M Preissner R Preissner S. The transformer database: biotransformation of xenobiotics. Nucleic Acids Res. (2014) 42:D1113–7. 10.1093/nar/gkt1246 24334957 PMC3965107

[27] Gaedigk A Riffel AK Berrocal BG Solaesa VG Dávila I Isidoro-García M. Characterization of a complex *CYP2D6* genotype that caused an AmpliChip *CYP450* Test® no-call in the clinical setting. Clin Chem Lab Med. (2014) 52:799–807. 10.1515/cclm-2013-0943 24445243

[28] Gaedigk A Hernandez J García-Solaesa V Sánchez S Isidoro-García M. Detection and characterization of {theCYP}2D6{*}9x2gene duplication in two Spanish populations: resolution of {AmpliChip} {CYP}450 test no-calls. Pharmacogenomics. (2011) 12:1617–22. 10.2217/pgs.11.107 22044417

[29] Lloret-Linares C Rollason V Lorenzini KI Samer C Daali Y Gex-Fabry M. Screening for genotypic and phenotypic variations in {CYP}450 activity in patients with therapeutic problems in a psychiatric setting, a retrospective study. Pharmacol Res. (2017) 118:104–10. 10.1016/j.phrs.2016.07.002 27378571

[30] Lu YF Goldstein DB Angrist M Cavalleri G. Personalized medicine and human genetic diversity. Cold Spring Harb Perspect Med. (2014) 4:a008581. 10.1101/cshperspect.a008581 25059740 PMC4143101

[31] König IR Fuchs O Hansen G von Mutius E Kopp M V. What is precision medicine? Eur Respir J. (2017) 50:1–12. 10.1183/13993003.00391-2017 29051268

[32] Beckmann JS Lew D. Reconciling evidence-based medicine and precision medicine in the era of big data: challenges and opportunities. Genome Med. (2016) 8:1–11. 10.1186/s13073-016-0388-7 27993174 PMC5165712

[33] Carrasco-Ramiro F Peiró-Pastor R Aguado B. Human genomics projects and precision medicine. Gene Therapy. (2017) 24:551–61. 10.1038/gt.2017.77 28805797

[34] Nofziger C Turner A Sangkuhl K Whirl-Carrillo M Agúndez J Black J. PharmVar GeneFocus: *CYP2D6*. Clin Pharmacol Ther. (2019) 107:154–70. 10.1002/cpt.1643 31544239 PMC6925641

[35] Botton M Whirl-Carrillo M Del Tredici A Sangkuhl K Cavallari L Agúndez J. PharmVar GeneFocus: *CYP2C19*. Clin Pharmacol Ther. (2020) 109:352–366. 10.1002/cpt.1973 32602114 PMC7769975

[36] Caudle K Sangkuhl K Whirl-Carrillo M Swen J Haidar C Klein T. Standardizing *CYP 2D6* genotype to phenotype translation: consensus recommendations from the clinical pharmacogenetics implementation consortium and dutch pharmacogenetics working group. Clin Transl Sci. (2019) 13:116–24. 10.1111/cts.12692 31647186 PMC6951851

[37] Taylor C Crosby I Yip V Maguire P Pirmohamed M Turner R. A review of the important role of *CYP2D6* in pharmacogenomics. Genes. (2020) 11:1295. 10.3390/genes11111295 33143137 PMC7692531

[38] Saravanakumar A Sadighi A Ryu R Akhlaghi F. Physicochemical properties, biotransformation, and transport pathways of established and newly approved medications: a systematic review of the top 200 most prescribed drugs vs. the FDA-approved drugs between 2005 and 2016. Clin Pharmacokin. (2019) 58:1281–94. 10.1007/s40262-019-00750-8 30972694 PMC6773482

[39] Gaedigk A Sangkuhl K Whirl-Carrillo M Twist GP Klein TE Miller NA. The Evolution of PharmVar. Clin Pharmacol Ther. (2019) 105:29–32. 10.1002/cpt.1275 30536702 PMC6312487

[40] Gaedigk A Whirl-Carrillo M Pratt VM Miller NA Klein TE. PharmVar and the landscape of pharmacogenetic resources. Clin Pharmacol Ther. (2020) 107:43–6. 10.1002/cpt.1654 31758698 PMC6925620

[41] Gaedigk A Ingelman-Sundberg M Miller NA Leeder JS Whirl-Carrillo M Klein TE. The pharmacogene variation (PharmVar) consortium: incorporation of the human cytochrome *P450* (*CYP*) allele nomenclature database. Clin Pharmacol Ther. (2018) 103:399–401. 10.1002/cpt.910 29134625 PMC5836850

[42] Gaedigk A Sangkuhl K Whirl-Carrillo M Klein T Leeder J. Prediction of *CYP2D6* phenotype from genotype across world populations. Genet Med. (2017) 19:69–76. 10.1038/gim.2016.80 27388693 PMC5292679

[43] De Leon J. AmpliChip *CYP450* test: personalized medicine has arrived in psychiatry. Expert Rev Mol Diagn. (2006) 6:277–86. 10.1586/14737159.6.3.277 16706732

[44] Seripa D Lozupone M Stella E Paroni G Bisceglia P La Montagna M. Psychotropic drugs and *CYP2D6* in late-life psychiatric and neurological disorders. What do we know? Expert Opin Drug Saf. (2017) 16:1373–85. 10.1080/14740338.2017.1389891 29025271

